# Follow-up after endoscopic resection for early gastric cancer in 3 French referral centers

**DOI:** 10.1016/j.igie.2022.10.004

**Published:** 2022-11-03

**Authors:** Bernadette de Rauglaudre, Mathieu Pioche, Fabrice Caillol, Jean-Philippe Ratone, Anna Pellat, Romain Coriat, Jerôme Rivory, Thomas Lambin, Laetitia Dahan, Marc Giovanini, Maximilien Barret

**Affiliations:** 1 Department of Digestive Endoscopy, Institut Paoli–Calmettes, Marseille, France; 2 Department of Gastro-enterology and Digestive Oncology, Hôpital Edourd Herriot and University of Lyon, Lyon, France; 3 Department of Gastro-enterology and Digestive Oncology, Hôpital de la Timone and University of Aix-Marseille, Marseille, France; 4 Department of Gastroenterology and Digestive Oncology, Hôpital Cochin and University of Paris Cité, Paris, France

## Abstract

**Background and aims:**

Endoscopic resection is the recommended staging and treatment modality for early gastric cancer (EGC). However, in Europe, data on long-term treatment outcomes and the occurrence of metachronous gastric cancer are lacking.

**Methods:**

Endoscopic resection for EGC was performed in 108 patients in 3 French referral centers. Resections were classified as eCuraA, B, C-1, or C-2 according to the new Japanese gastric cancer treatment guidelines and European guidelines. Patients who did not undergo secondary surgery and had follow-up data after 6 months were included in the analysis.

**Results:**

One hundred eight patients underwent endoscopic resection for EGC. Surgery was performed in 32 patients after endoscopic resection. Forty-five patients had follow-up visits at ≥6 months: 27 with an eCuraA resection, 1 with an eCuraB resection, 6 with an eCuraC-1 resection, and 11 with an eCuraC-2 resection. During a median follow-up time of 29.39 months, metachronous recurrence was observed in 12 patients (12/45; 26.7%) with a median period of 15.43 months (range, 7-44). Metastatic recurrence was observed in 3 patients (3/45, 6.7%), with a median period of 28.91 months (range, 7.52-28.91). Morbidity and mortality at day 30 after endoscopic resection were 2.8% (3/108) and 0.9% (1/108), respectively. Metachronous recurrences were diagnosed in 18.5% of the eCuraA group, 0% of the eCuraB group, 33.3% of the eCura C-1 group, and 45.5% of the eCura C-2 group; the differences were not statistically significant (*P* = .061).

**Conclusion:**

The rate of metachronous recurrence after endoscopic resection of early gastric cancer was very high, even after a curative resection. Close endoscopic surveillance is necessary to diagnose and treat metachronous gastric cancer.

As the incidence and mortality rate of gastric cancer have steadily declined,^1^ the number of patients diagnosed with early gastric cancer (EGC) has increased during the past few years because of recent technical advances in endoscopic assessment.^2^

Endoscopic resection is now accepted as a treatment option for EGC, according to the new Japanese gastric cancer treatment guidelines^3^ and European guidelines.^4^ For patients who meet the absolute and expanded criteria, endoscopic resection can be performed en bloc with negligible risk of lymph node metastasis.^5^, ^6^, ^7^, ^8^ Endoscopic resection has shown excellent outcomes and safety.^9^ In Asia, the cumulative incidence of metachronous recurrences after endoscopic resection of EGC ranges from 2.7% to 15.6%.^10^, ^11^, ^12^, ^13^ However, most studies in the West focus on endoscopic therapy and early outcomes, and the rate of metachronous recurrences in the long term is largely unknown, mostly owing to the lower incidence of EGCs in Europe and the smaller sizes of the studies.^14^^,^^15^ In addition, there is an ongoing debate about whether the Asian resection criteria can be used in European and other non-Asian patients.^16^

We aimed to determine the outcomes of endoscopic resection for early gastric cancer.

## Patients and methods

The study was conducted in 3 French referral centers: Paoli–Calmettes Institute (Marseille), Cochin Hospital (Paris), and Edouard Heriot Hospital (Lyon). The study was approved by the clinical research and innovation department of the Paoli–Calmettes Institute (IRB: METASTOMACH-IPC 2021-080, approval on November 11, 2021).

### Inclusion and exclusion criteria

All patients referred to the Paoli-Calmettes Institute from January 2010 to October 2020 and to the Cochin and Edouard Heriot hospitals from January 2016 to October 2020 for endoscopic resection of EGC were included. Data were collected retrospectively.

We analyzed the clinical data of 108 patients aged >20 years who underwent endoscopic resection of the EGC (including lesions of the gastric cardia) with EMR, endoscopic submucosal dissection (ESD), or hybrid technique. We excluded patients who met any of the following criteria: (1) neuroendocrine lesion or intraepithelial dysplasia (low grade or high grade), (2) history of previous abdominal surgery for stomach cancer, (3) surgery performed immediately after endoscopic resection, or (4) follow-up time of <6 months after endoscopic resection.

Relevant clinical data were extracted, including patient demographics, lesion characteristics, procedural details, procedure-related adverse events, treatment outcomes, metachronous and metastatic recurrences, and vital status at the last follow-up visit, when available.

### Definitions, treatment decision after endoscopic resection, and follow-up

The diagnosis of EGC was made in the referral center or at another institution, and the patients were referred for resection. After the diagnostic work-up, endoscopic resection was performed. The choice of the endoscopic resection technique was made by the endoscopist.

After resection, the histopathologic diagnosis and decisions about further treatment were discussed by a local multidisciplinary board. Well and moderately differentiated cancers were considered differentiated-type lesions, and poorly differentiated adenocarcinoma and signet-ring cell carcinoma were considered undifferentiated-type lesions. En bloc resection was defined as resection of the targeted area in 1 piece. R0 or histologically complete resection was defined by neoplasia-free vertical and horizontal margins. Resection was considered as follows:

Resection eCuraA was considered for an en bloc resection with negative horizontal and vertical margins and no lymphovascular invasion (LVI), and a lesion with these characteristics:
  <2 cm in diameter, predominantly differentiated type, pT1a, and nonulcerated
  ≥2 cm in diameter, predominantly differentiated type, pT1a, and nonulcerated
  <3 cm, predominantly differentiated type, pT1a, and ulcerated
  <2 cm, predominantly undifferentiated type, pT1a, and nonulcerated
Resection eCuraB was considered for an en bloc resection with negative horizontal and vertical margins and no lymphovascular invasion (LVI), lesions <3 cm, predominantly differentiated type, pT1b ≤500 μm (SM1), and nonulcerated.
Resection eCuraC-1 was considered for differentiated-type lesions that fulfilled other criteria to be classified into either eCuraA or eCuraB but was either not resected en bloc or had positive horizontal margins (R1).
Resection eCuraC-2 was considered for all other lesions.

In the case of positive margins at the horizontal margin (eCuraC-1), endoscopic follow-up was performed, and further endoscopic resection was performed as needed. Patients with eCuraC-2 resection were offered surgery after a multidisciplinary evaluation.

Endoscopic follow-up was scheduled at 3 months, 6 months, 12 months, and every year after endoscopic resection if surgery was not performed.

Observation began on the day of endoscopic resection and was stopped in cases of recurrence or death. The following data were retrieved from the medical file or by phone call. Local recurrence was defined as gastric recurrence at the resection site. Metachronous recurrence was defined as gastric recurrence away from the resection site. An unclearly localized gastric recurrence occurring only after 6 months and after 1 endoscopic follow-up visit without recurrence was considered metachronous also. Metastatic recurrence was defined as recurrence distant from the gastric cancer lesion (eg, lymph node, liver, lung metastasis).

Disease-free survival (DFS) was defined as the period from the initial endoscopic resection until recurrence or death.

### Safety of endoscopic treatment

Morbidity and mortality were graded according to the AGREE classification.^16^ Morbidity was defined as any severe procedure-related adverse event (AGREE ≥2) within 30 days after endoscopic resection. Mortality was defined as any procedure-related death within 30 days after endoscopic resection.

Severe intraprocedural bleeding was defined as clinical bleeding with a drop in hemoglobin >2 g/dL and/or with the need for blood transfusion. Transmural perforation was defined as an obvious endoscopic view into the peritoneal cavity or when postinterventional imaging showed pneumoperitoneum and/or effusion in a symptomatic patient.

### Outcomes

The primary endpoint was the rates of recurrence, including metachronous recurrence and metastatic recurrence. Secondary outcomes included DFS, gastric cancer–related death, and rates of adverse events. Lesions were classified as eCuraA, eCuraB, eCuraC-1, or eCuraC-2 according to the histologic features of the resection specimen.

### Statistical analysis

Frequencies and percentages were calculated as categoric variables. The mean, standard deviation (SD), median, and interquartile range (IQR) were calculated as continuous variables.

Kaplan-Meier curves were created, and log-rank regression analysis was performed to compare the DFS of the different groups. *P* values <.05 were considered statistically significant. All calculations were performed with GraphPad Prism 9 (GraphPad Software, San Diego, Calif, USA).

## Results

### Patient characteristics

A total of 108 patients underwent endoscopic resection for EGC. The clinicopathologic characteristics of the patients who underwent endoscopic resection are shown in Table 1.

**Table 1:** Patient and lesion baseline characteristics

Patient characteristics (N = 108)	
Sex, male/female, N (%)	70 (64.8%)/38 (35.2%)
Age, median (range)	71.8 (28.6–92.1)
BMI, median (range)	24.8 (16.5–38.3)
ASA status 1/2/3/4/NA, N (%)	13 (12%)/50 (46.3%)/24 (22.2%)/1 (0.9%) /20 (18.5%)
Lesion characteristics and procedure	
Type of resection: EMR/ESD/Hybrid, n (%)	29 (26.9%)/64 (59.3%)/15 (13.9%)
Tumor location, n (%)	
Gastric cardia	23 (21.3%)
Stomach	85 (78.7%)
En bloc resection, n (%)	87 (80.6%)
Tumor size, mm, n (%)	
≤ 20	49 (45.4%)
21–30	30 (27.8%)
> 30	24 (22.2%)
NA	5 (4.6%)
Hospitalization time, median (range)	3 (0–35)
Resection outcomes	
Invasion depth, n (%)	
Mucosal, pT1a	70 (64.8%)
Submucosal, pT1b ≤ 500 μm	3 (2.8%)
Submucosal, pT1b > 500 μm	33 (30.6%)
NA	2 (1.9%)
Differentiation status, n (%)	
Differentiated	78 (72.2%)
Undifferentiated/signet-ring cells	23 (21.3%)
NA	7 (6.5%)
Lymphovascular invasion, n (%)	14 (13.0%)
Positive margins, n (%)	20 (18.5%)
Vertical margins	18 (16.7%)
Horizontal margins	22 (20.4%)

*ASA*, Physical status score; *BMI*, body mass index; *EMR*, endoscopic mucosal resection; *ESD*, endoscopic submucosal dissection; *NA*, not applicable.

Thirty-two patients were excluded because they underwent surgery after endoscopic resection: 27 in the eCuraC-2 group, 4 in the eCuraC-1 group, and 1 in the eCuraA group. Among these 32 patients who underwent surgery immediately after endoscopic resection, 11 had no residual cancer at the endoscopic resection site, 10 had residual cancer at the endoscopic resection site, 5 had locoregional lymph node metastasis, and 6 had surgery for which we do not have the anatomopathologic results. Thirteen patients in the eCuraC-2 and eCuraC-1 groups did not undergo surgery because of patient refusal or comorbidity.

Repeated endoscopic resection was performed in 5 patients, for horizontal margins (eCuraC-1, n = 3) or clinician’s decision (eCuraC-2, n = 2). All of the repeated endoscopic resections resulted in a curative resection (eCuraA).

**Figure 1:**
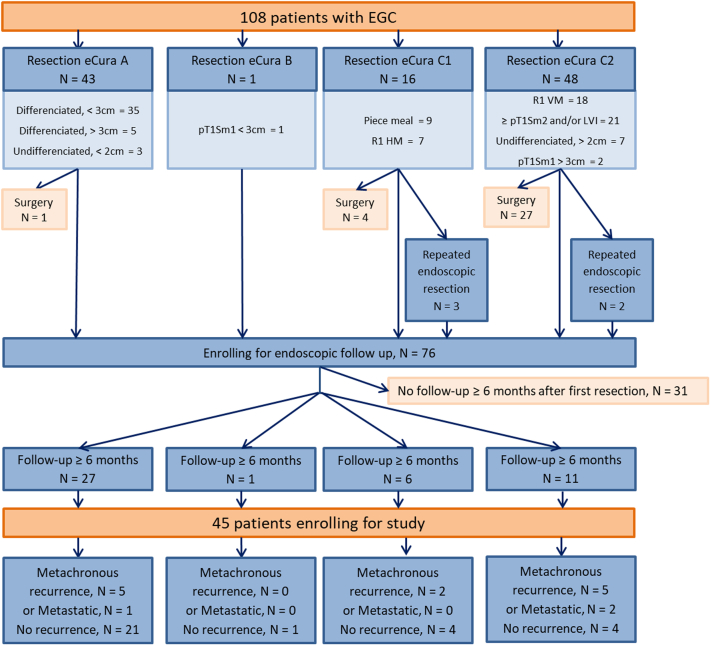
Study flowchart. *EGC*, Early gastric cancer; *HM*, horizontal margin; *LVI*, lymphovascular invasion; *VM*, vertical margin.

A total of 76 patients were followed up. Thirty-one patients had a follow-up time of <6 months and were excluded from the analysis. Among these 31 patients, 1 had local recurrence after an eCuraC-1 resection (1/3), and 2 had a synchronous cancer, not seen at the first endoscopic examination after an eCuraA resection (2/3).The study flowchart is presented in Figure 1.

### Follow-up

Forty-five patients had a follow-up time ≥6 months and were analyzed. The clinicopathologic characteristics of the patients are shown in Table 2.

**Table 2:** Patients with follow-up ≥6 months (N = 45)

Variable	eCuraA (n = 27)	eCuraB (n = 1)	eCuraC-1 (n = 6)	eCuraC-2 (n = 11)
Type of resection				
EMR/ESD/hybrid, n	7/20/1	0/1/0	0/6/0	1/8/2
En bloc resection, n	27	1	2	10
Tumor size, mm, n				
≤20	14	1	1	6
21-30	9	-	2	3
>30	4	-	3	2
NA	-	-	-	-
Invasion depth, N				
Mucosal, pT1a	27	-	6	3
Submucosal, pT1b ≤500 μm	-	1	-	1
Submucosal, pT1b >500 μm	-	-	-	7
NA	-	-	-	-
Differentiation status, n				
Differentiated	21	1	5	7
Undifferentiated	3	-	1	3
NA	3	-	-	1
Lymphovascular invasion, n	-	-	-	2
Positive margins, n				
Vertical margins	-	-	-	-
Horizontal margins	-	-	2	2
Time of follow-up, months, median (range)	28.93 (6.02-102.51)	39.98	44.32 (14.10-59.44)	12.00 (6.94-46.29)
Death, n				
Gastric cancer	1	-	-	2
Other causes	1	-	-	1
Metachronous recurrence	5	-	2	5
Distant recurrence	1	-	-	2
Time after initial resection, months, median (range)	28.48 (16.76-38.73)	-	28.83 (14.10-43.56)	10.74 (6.93-26.74)
Treatment of recurrence				
Endoscopic resection	3	-	2	4
Surgery	1	-	-	1
Chemotherapy	1	-	-	-
Palliative care	-	-	-	1

*ESD*, Endoscopic submucosal dissection; *EMR*, endoscopic mucosal resection; *NA*, not applicable.

During a median follow-up period of 29.39 months (range, 6.02-102.51 months, 0.50-8.54 years), recurrence was observed in 15 patients (15/45, 33.3%). Recurrences were diagnosed in a median time of 15.43 months (range, 7-44 months, 0.58-3.63 years).

Metachronous recurrences were observed in 12 patients (12/45; 26.7%) in a median period of 15.43 months (range, 7-44 months, 0.58-3.63 years). All patients with metachronous recurrences had an unremarkable first follow-up endoscopy result. In addition, metachronous recurrences were observed after an ESD in 9 of 12 (75%) patients, after an initial R0 resection in 10 of 12 (83.3%) patients, and after an en bloc resection in 10 of 12 (83.3%) patients. Metachronous recurrences were treated by endoscopic resection (10/12) or surgery (2/12), with curative resections in all cases.

**Figure 2:**
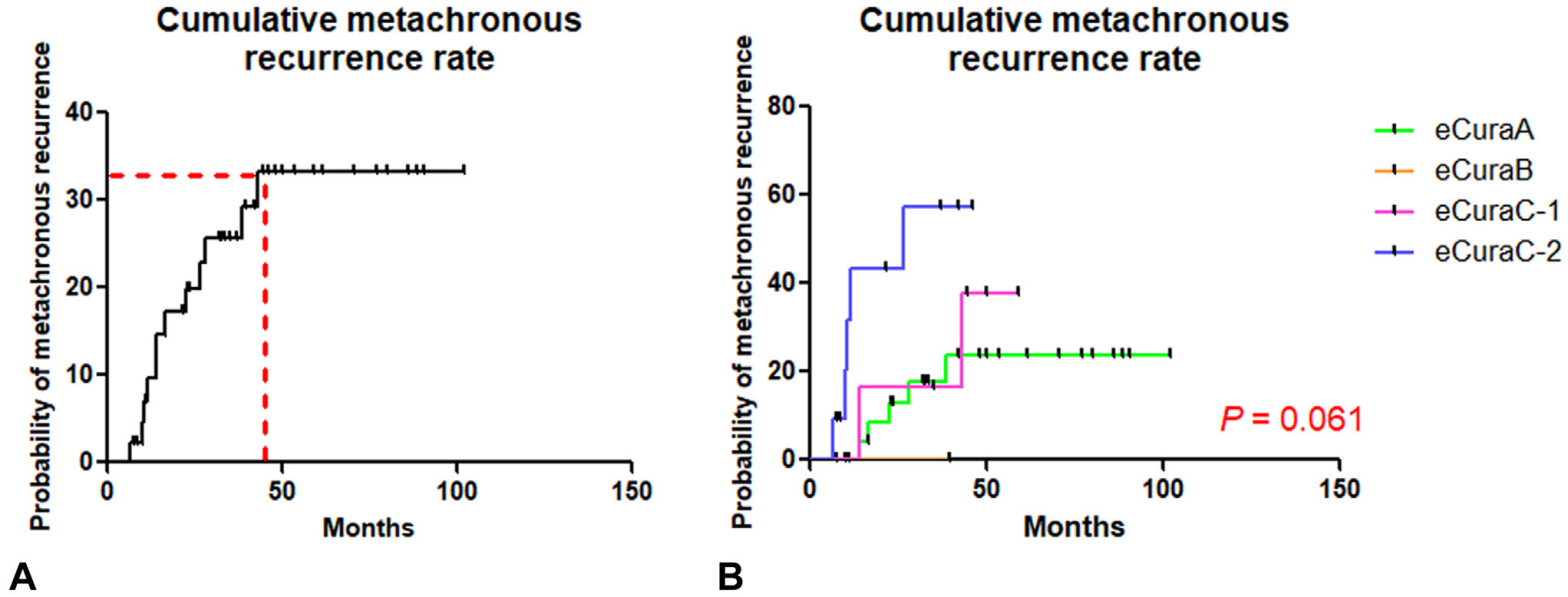
Kaplan-Meier curve for cumulative metachronous recurrence rates for the study population. **A,** 45 patients included in the analysis. **B,** Patients in each eCura group.

Metachronous recurrence during the follow-up period was diagnosed in 5 of 27 (18.5%) of the eCuraA group, 0 of 6 patients of the eCuraB group, 2 of 6 (33.3%) of the eCuraC-1 group, and 5 of 11 (45.5%) of the eCuraC-2 group without statistically significant differences among the 4 groups (*P* = .061). Figure 2 shows the Kaplan-Meier curve for cumulative metachronous recurrence rates after endoscopic resection.

No local recurrence was observed during the follow-up period up to 6 months.

Metastatic recurrence was observed in 3 patients (3/45; 6.7%) in a median period of 28.91 months (7.52-28.91 months, 0.63-2.80 years). One patient received palliative chemotherapy for unresectable disease (with linitis), and 2 patients underwent supportive care because they were poor candidates for any intervention (1 with distant metastasis and 1 with gastric stenosis). Metastatic recurrence was observed in 1 patient (1/27, 3.7%) of the eCuraA group and 2 patients (2/11, 18.2%) of the eCuraC-2 group.

A total of 30 patients had no recurrence during the follow-up period (30/45, 66.7%). For patients who had no recurrence during the follow-up period, endoscopy showed low-grade dysplasia in 4 patients (4/30, 13%) and metaplasia in 9 patients (9/30, 30%).

### Safety of endoscopic procedure

The 30-day morbidity and mortality after endoscopic resection were 2.8% (3/108) and 0.9% (1/108), respectively. Bleeding was recorded in 2 patients who needed transfusion and prolonged hospital admission. These adverse events were classified as AGREE II. Gastric perforation occurred in 1 patient and was managed endoscopically but with intensive care admission. This adverse event was classified as AGREE IVa. A patient died of a multivisceral failure secondary to aspiration pneumonia during anesthesia, 10 days after endoscopic resection. This adverse event was classified as AGREE V.

## Discussion

We conducted a retrospective cohort study to investigate outcomes after endoscopic resection for EGC. In our study, 108 patients underwent endoscopic resection for EGC. En bloc resection was achieved in 80.6% and R0 resection in 80.6% of all lesions. Morbidity was 2.8%. Altogether, endoscopic resection of EGC appeared to be safe and less morbid than surgery.^9^^,^^17^ Among patients with >6 months of follow-up care, metachronous recurrences occurred in 26.7% of patients, and metastatic recurrence occurred in 6.7%. Noticeably, 4 patients with an initial eCuraC-2 resection (4/11, 36.4%) did not present with recurrence during the follow-up period, which suggests that endoscopic resection could be an option for inoperable patients.^18^^,^^19^

In our study, only 40.7% of all resections fulfilled the Japanese criteria for curative resection (eCuraA or eCuraB, n = 44), and 59.3% of the resected lesions were considered noncurative (eCuraC-1 or eCuraC-2, n = 64). Probst et al^14^ also found a high (22.8%) rate of noncurative resections in a large study including 179 patients with 191 EGCs. Indeed, in the absence of screening endoscopies, gastric cancer is more likely to be diagnosed at more advanced stages in Western countries.^20^ Because of the low number of Western patients included in the currently published studies and because carcinogenesis may differ between ethnic groups,^21^ extrapolation of Japanese recommendations to Western patients has been debated.^19^ Indeed, the frequency of lymph node metastasis in patients with pT1 may differ from that in Eastern Asian and Western countries. More broadly, race has been reported as an independent risk factor for lymph node metastasis.^22^ In Asian countries, the risk of lymph node metastasis was estimated to be 2.7% for mucosal carcinomas (ie, T1a) and 22.9% for submucosal carcinomas (ie, T1b)^23^ compared with the 6.5% to 13% and 21% to 23.9% risks, respectively, in Europe.^24^, ^25^, ^26^ This underlines the need for a maximum amount of data from Western patients with EGC.

We found a 26.7% rate of metachronous recurrence, higher than the 9.3% to 18.4% reported in the literature with a similar follow-up times.^11^^,^^13^^,^^14^^,^^27^, ^28^, ^29^, ^30^, ^31^, ^32^ We did not find a statistically significant difference related to the curability of the resection (eCura groups), but we found a trend toward significance (*P* = .061), and this may become significant with larger numbers of patients. Patients, especially those with noncurative resection (eCuraC-1 or C-2), should undergo resection surgery whenever possible, or a careful and regular endoscopic follow-up. The risk of metachronous recurrence has important clinical implications for patients with EGC who are followed up after endoscopic resection, underlining the importance of periodic and long-term endoscopic follow-up. Follow-up after endoscopic resection should be performed approximately 6 months after endoscopic resection,^33^ followed by annual prolonged endoscopic follow-up times^10^^,^^27^^,^^34^ and ongoing oncologic surveillance as well (ie, cross-sectional imaging) for diagnosis of metastatic recurrence.^3^ In addition, the gastroenterologist needs to inform the patient of the risk of recurrence.

An important issue in our study was the definition of metachronous recurrence. Because of the retrospective design, the localization of the recurrence was often uncertain in the endoscopic records. To minimize this imprecision in the data, we excluded early recurrences (<6 months) and patients with follow-up times <6 months. It is a probably a limitation of the study, but this choice is supported by the high rate of gastric relapse after curative resection, which cannot be anything else other than metachronous lesions. In addition, most metachronous recurrences followed R0 resections, ruling out confusion with local recurrence. Metachronous recurrence is explained by the fact that only the EGC lesion is removed, without ablation of the remaining atrophic or metaplastic gastric mucosa, similar to resection of an early esophageal adenocarcinoma without ablating the remaining Barrett’s esophagus.^35^

The strengths of our study were the inclusion of a significant number of patients with invasive gastric cancer resected endoscopically, with significant follow-up data only, and the multicentric study design.

Other limitations of our study were the retrospective design and the number of patients lost to follow-up care. The missing follow-up data can be explained by the decision by some patients to be followed up at an external institute, principally owing to the distance from the referral center. A major limitation of our study was the absence of data about *Helicobacter pylori* (HP) status and presence or absence of atrophy, owing to a large amount of missing data. This bias must be taken into account in the interpretation of these results. HP infection is an independent risk factor for the subsequent development of cancer after endoscopic resection of an EGC lesion, and its eradication drastically reduces the risk of recurrence.^36^^,^^37^ The rate of R0 at the first resection was lower than that in other studies,^10^^,^^14^ which should also be acknowledged as a limitation of our work. This is explained by the length of the inclusion period, leading to the inclusion of several patients treated with piecemeal EMR.

It seems important to understand the risk factors for metachronous recurrence after endoscopic resection. Generally, the known factors related to metachronous recurrences are male sex, age, smoking, tumor size, HP infection, histologic features, invasion depth, and curative resection.^10^^,^^38^ The number of patients included in our study was insufficient to enable us to determine the risk factors in patients for metachronous recurrence.

## Conclusion

In conclusion, although endoscopic resection of early gastric cancer is safe and effective, the rate of metachronous early gastric cancer is high, even after curative resection, and warrants regular and prolonged endoscopic follow-up.

## Disclosure

The following authors disclosed financial relationships: J. Rivory: Consultant for Cook and Olympus. M. Barret: Board participation in Norgine and Ambu; Research grant from Pentax. All other authors disclosed no financial relationships.
